# Ca-*α*1T, a fly T-type Ca^2+^ channel, negatively modulates sleep

**DOI:** 10.1038/srep17893

**Published:** 2015-12-09

**Authors:** Kyunghwa Jeong, Soyoung Lee, Haengsoo Seo, Yangkyun Oh, Donghoon Jang, Joonho Choe, Daesoo Kim, Jung-Ha Lee, Walton D. Jones

**Affiliations:** 1 KAIST, Department of Biological Sciences, Daejeon, 305-701, Republic of Korea; 2 Sogang University, Department of Life Sciences, Seoul, 121-742, Republic of Korea

## Abstract

Mammalian T-type Ca^2+^ channels are encoded by three separate genes (Ca_v_3.1, 3.2, 3.3). These channels are reported to be sleep stabilizers important in the generation of the delta rhythms of deep sleep, but controversy remains. The identification of precise physiological functions for the T-type channels has been hindered, at least in part, by the potential for compensation between the products of these three genes and a lack of specific pharmacological inhibitors. Invertebrates have only one T-type channel gene, but its functions are even less well-studied. We cloned Ca-*α*1T, the only Ca_v_3 channel gene in *Drosophila melanogaster*, expressed it in *Xenopus* oocytes and HEK-293 cells, and confirmed it passes typical T-type currents. Voltage-clamp analysis revealed the biophysical properties of Ca-*α*1T show mixed similarity, sometimes falling closer to Ca_v_3.1, sometimes to Ca_v_3.2, and sometimes to Ca_v_3.3. We found Ca-*α*1T is broadly expressed across the adult fly brain in a pattern vaguely reminiscent of mammalian T-type channels. In addition, flies lacking Ca-*α*1T show an abnormal increase in sleep duration most pronounced during subjective day under continuous dark conditions despite normal oscillations of the circadian clock. Thus, our study suggests invertebrate T-type Ca^2+^ channels promote wakefulness rather than stabilizing sleep.

T-type Ca^2+^ channels are a subfamily of voltage-dependent Ca^2+^ channels (VDCCs) that produce low-voltage-activated (LVA) Ca^2+^ currents implicated in NREM sleep in mammals^1^. Three different genes encode the pore-forming alpha subunits of mammalian T-type channels, Ca_v_3.1, 3.2, and 3.3. Of these, Ca_v_3.1 and 3.3 are highly expressed in the thalamus, where the oscillations required for NREM sleep are generated^2^,^3^,^4^. Mice lacking Ca_v_3.1 show reduced delta-wave activity and reduced sleep stability, suggesting that mammalian T-type currents have a sleep-promoting or stabilizing function^1^.

Unlike mammals, *Drosophila melanogaster* has only one T-type Ca^2+^ channel, Ca-*α*1T, which is also known as Dm*α*G. A recent study found that motor neurons in flies lacking Ca-*α*1T show reduced LVA but also reduced high-voltage-activated (HVA) Ca^2+^ currents, suggesting that although Ca-*α*1T seems to be a genuine T-type channel, it may have interesting biophysical properties^5^. We therefore cloned a single isoform of Ca-*α*1T, expressed it in *Xenopus* oocytes or HEK-293 cells, and compared its biophysical properties with those of the rat T-type channel Ca_v_3.1. We also generated several Ca-*α*1T mutant alleles and identified a defect in their sleep/wake cycles. Contrary to results in mammals, the fly T-type Ca^2+^ channel destabilizes sleep. We anticipate that our findings will help clarify species-dependent differences in the *in vivo* functions of T-type Ca^2+^ channels, particularly their role in sleep physiology.

## Results

### Ca-*α*1T produces LVA currents in *Xenopus* oocytes

The fly T-type Ca^2+^ channel Ca-*α*1T spans roughly 90 kilobases of genomic DNA and has five different annotated mRNA transcripts designated RB through RF. Because the smallest of these transcripts is still over 10 kilobases in length, we used a piece-meal approach to assemble a full-length cDNA. To do so, we isolated total RNA from fly heads and used reverse transcription to produce cDNAs. Using these cDNAs as a template, we amplified and then assembled partial clones to obtain full-length cDNAs for both the RB (NM_132068) and RC (NM_001103419) Ca-*α*1T transcripts. After sequence verification, we used these Ca-*α*1T cDNA clones to produce cRNAs for injection into *Xenopus* oocytes. We were able to confirm expression of the RC isoform, but not the RB isoform, by measuring robust inward currents using 10 mM Ba^2+^ as a charge carrier 4 days after cRNA injection. In all subsequent experiments performed with this RC isoform cDNA, we refer to it simply as Ca-*α*1T.

**Figure 1:**
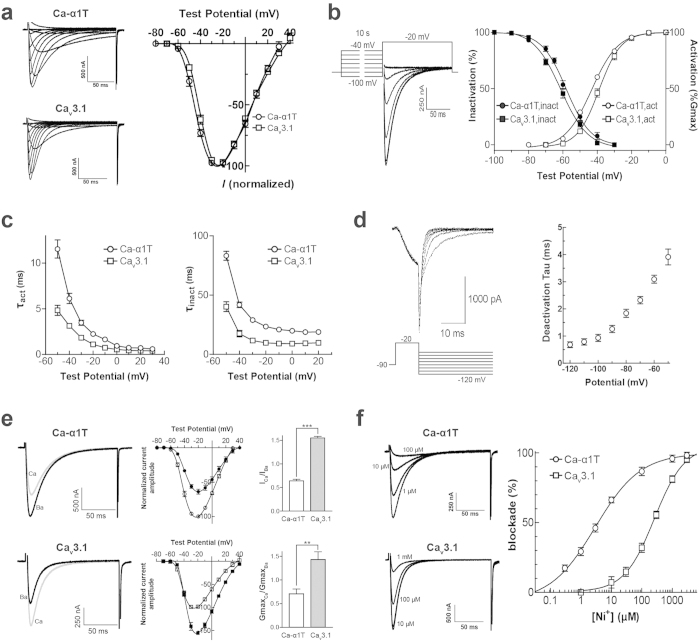
Comparing the biophysical properties of Ca-*α*1T and rat Ca_v_3.1. (**a**) (Left) Representative current traces through Ca-*α*1T and Ca_v_3.1 expressed in *Xenopus* oocytes. In 10 mM Ba^2+^, currents were elicited by depolarizing 10 mV step pulses (−70 mV to +40 mV) from a holding potential of −90 mV. (Right) I–V relationships of Ca-*α*1T and Ca_v_3.1. Peak currents for each oocyte were normalized to the maximum current. Percent amplitudes from oocytes expressing Ca-*α*1T (○) or Ca_v_3.1 (⌜) plotted against test potentials and fitted with the Boltzmann equation. (**b**) (Left) Steady-state inactivation measured during voltage steps to −20 mV after 10 s prepulses to potentials between −100 mV and −40 mV. (Right) Voltage-dependent activation and steady-state inactivation curves of Ca-*α*1T (○, ●) and Ca_v_3.1 (⌜, ■) fitted to the Boltzmann equation. (**c**) The activation (*τ*_act_) and inactivation (*τ*_inact_) time constants for Ca-*α*1T (○) and Ca_v_3.1 (⌜) obtained by fitting the current traces to double exponentials. (**d**) Voltage-dependent deactivation of Ca-*α*1T in HEK-293 cells. Tail currents elicited by step pulses to −20 mV for 10 ms, followed by re-polarizing potentials (−120 mV to −50 mV). Deactivation time constants were obtained by fitting the traces to a single exponential and plotted against re-polarizing potentials. (**e**) I_Ca_/I_Ba_ ratios of Ca-*α*1T and Ca_v_3.1. (Left) Representative current traces through Ca-*α*1T and Ca_v_3.1 measured in 10 mM Ba^2+^ or 10 mM Ca^2+^ elicited by 10 mV step pulses from a holding potential of −90 mV. Ba^2+^ currents are black; Ca^2+^ currents are grey. (Middle) I–V relationships of Ca-*α*1T (○, ●) and Ca_v_3.1 (⌜, ■) in 10 mM Ba^2+^ (open) or 10 mM Ca^2+^ (filled). (Right) Peak current ratios (I_Ca_/I_Ba_) and relative slope conductance (G_MaxCa_/G_MaxBa_) for Ca-*α*1T and Ca_v_3.1. Student’s t-test, **p < 0.01, ***p < 0.001. (**f**) Nickel inhibition sensitivity of Ca-*α*1T and Ca_v_3.1. (Left) Representative current traces of Ca-*α*1T and Ca_v_3.1 at various Ni^2+^ concentrations. (Right) Dose-response curves indicating Ni^2+^-dependent inhibition of Ca-*α*1T (○) and Ca_v_3.1 (⌜). Data are presented as means ± s.e.m.

We next compared the biophysical properties of Ca-*α*1T with those of a mammalian T-type Ca^2+^ channel homolog, rat Ca_v_3.1^6^, using the *Xenopus* oocyte expression system. Both Ca-*α*1T and Ca_v_3.1 have low-voltage activation thresholds, but the threshold of Ca-*α*1T (−60 mV) is slighty lower than that of the rat channel by 3 ~ 4 mV. Both Ca-*α*1T and Ca_v_3.1 produce current kinetics typical of T-type Ca^2+^ channels when subjected to a protocol of serial step pulses from a holding potential of −90 mV. Although the inactivation kinetics of Ca-*α*1T are slightly slower than those of Ca_v_3.1, both the activation and inactivation kinetics of currents through Ca-*α*1T accelerate at higher step pulse values. This produces the criss-crossing pattern typical of T-type Ca^2+^ channels (Fig. 1a). Together, these biophysical properties—an activation threshold of −60 mV, a potential of maximal current at −20 mV, transient current kinetics, a criss-crossing pattern in currents evoked by a step pulse voltage protocol—all of these properties mark Ca-*α*1T as a typical T-type Ca^2+^ channel^6^,^7^,^8^,^9^.

We next obtained activation curves for Ca-*α*1T and Ca_v_3.1 by fitting chord conductances with the Boltzmann equation. The potential for half-maximal activation (V_50,act_) of Ca-*α*1T and Ca_v_3.1 are −43.32 ± 1.58 and −38.92 ± 1.15 mV, respectively. This indicates that Ca-*α*1T is activated at 4.4 mV lower test potentials than Ca_v_3.1 (*p* < 0.05, Student’s t-test) (Fig. 1b and Table 1). During steady-state inactivation, the potentials of 50% channel availability (V_50, inact_) for Ca-*α*1T and Ca_v_3.1 are estimated to be −58.04 ± 0.71 and −61.31 ± 0.70 mV (*p* < 0.05, Student’s t-test). In other words, the V_50,inact_ of Ca-*α*1T is 3.3 mV more positive than that of Ca_v_3.1 (Fig. 1b and Table 1). An ion channel’s so-called “window current” is the range of overlap in its activation and steady-state inactivation curves. This window for Ca-*α*1T is considerably larger than that of Ca_v_3.1, implying that Ca-*α*1T is capable of persistently passing larger currents over the relevant voltage range than Ca_v_3.1.

The voltage-dependent kinetics of the three mammalian T-type calcium channels are known to differ, with Ca_v_3.1 and Ca_v_3.2 showing faster activation/inactivation kinetics than Ca_v_3.3^10^. To compare the time constants of activation and inactivation for Ca-*α*1T and Ca_v_3.1, we fitted current traces with a double exponential function. At test potentials ranging from −50 mV to +20 mV, Ca-*α*1T has slower current kinetics than Ca_v_3.1 (*p* < 0.01 for *τ*_act_ and *p* < 0.001 for *τ*_inact_, Student’s t-test, Fig. 1c). For example, the activation and inactivation time constants of Ca-*α*1T current at a −20 mV test potential are 2.2 ± 0.2 ms and 23.4 ± 1.4 ms respectively, whereas the activation and inactivation time constants of Ca_v_3.1 current at the same test potential are 1.1 ± 0.2 ms and 9.7 ± 0.9 ms, respectively. This means the activation and inactivation kinetics of Ca-*α*1T are 2-fold slower than those of rat Ca_v_3.1, but still in the “fast” range (Table 1).

Another defining property of the LVA T-type calcium channels is that they deactivate much more slowly than HVA calcium channels^6^,^9^,^11^. To characterize the deactivation kinetics of Ca-*α*1T, we performed a transient transfection of the Ca-*α*1T cDNA into HEK-293 cells followed by whole-cell patch clamp recordings of tail currents. As expected, the tail currents of Ca-*α*1T appear to undergo a slow voltage-dependent decay (Fig. 1d). The deactivation time constant obtained for Ca-*α*1T (0.93 ± 0.14 ms) by curve-fitting the tail currents is in the same range as that reported for all three mammalian Ca_v_3 isoforms (Table 1).

Previous studies have shown that Ca_v_3.1 passes larger amplitude currents when Ca^2+^ is used as a charge carrier rather than equimolar Ba^2+^, but that the opposite is true for Ca_v_3.2 and Ca_v_3.3^12^,^13^. We, thus, measured the relative permeability of Ca-*α*1T and Ca_v_3.1 to Ca^2+^ and Ba^2+^ ions (I_Ca_/I_Ba_). Consistent with previous reports, we measured a I_Ca_/I_Ba_ ratio for Ca_v_3.1 of 1.55 ± 0.03. This means the peak current amplitude of Ca_v_3.1 is greater in 10 mM Ca^2+^ than 10 mM Ba^2+^ (Fig. 1e). The I_Ca_/I_Ba_ ratio for Ca-*α*1T, however, is 0.68 ± 0.04. This means Ca-*α*1T passes a smaller current in 10 mM Ca^2+^ than in 10 mM Ba^2+^ (Fig. 1e). We also compared the relative permeability of Ca-*α*1T and Ca_v_3.1 to these two divalent cations by comparing their maximal slope conductance ratios (G_MaxCa_/G_MaxBa_). The G_MaxCa_/G_MaxBa_ ratios for Ca-*α*1T and Ca_v_3.1 are 0.71 ± 0.10 (n = 6) and 1.43 ± 0.17 (n = 4), respectively (Fig. 1e). Thus, in terms of its relative permeability to Ca^2+^ and Ba^2+^, Ca-*α*1T is more similar to Ca_v_3.2 or Ca_v_3.3 than Ca_v_3.1 (Table 1).

Finally, T-type channel isoforms are also known to be differentially sensitive to blockage by Ni^2+^ ions, with Ca_v_3.2 being the most sensitive of the three Ca_v_3 isoforms^14^. Low micromolar levels of Ni^2+^ produce a concentration-dependent inhibition of Ca-*α*1T (IC_50_ = 5.12 *μ*M), while much higher levels of Ni^2+^ are necessary for blockage of Ca_v_3.1 (IC_50_ = 276.5 *μ*M) (Fig. 1f). Thus, in terms of Ni^2+^ sensitivity, Ca-*α*1T more closely resembles Ca_v_3.2 than Ca_v_3.1 (Table 1).

### Ca-*α*1T is broadly expressed in the adult brain

**Figure 2:**
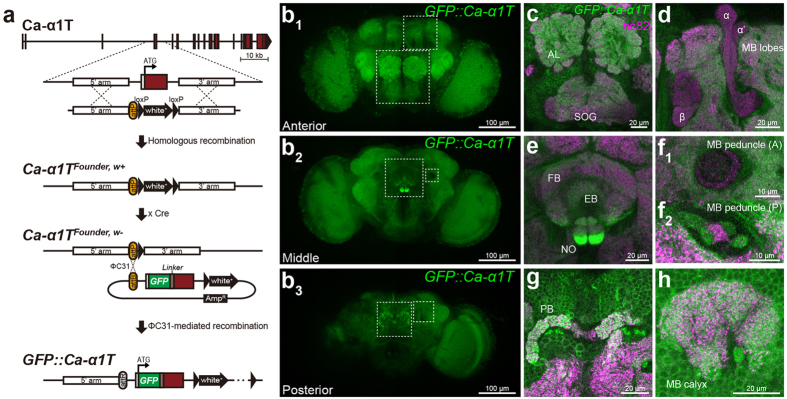
GFP::Ca-*α*1T expression in the adult brain. (**a**) Gene targeting and *GFP::Ca-α1T* generation strategy. Ca-*α*1T coding exons are red. (**b**) Adult brain expression of *GFP::Ca-α1T* (green) divided into maximal intensity projections of confocal stacks from the anterior (**b1**), middle (**b2**), and posterior (**b3**) brain. (**c–h**) GFP::Ca-*α*1T expression in specific neuropils whose location corresponds to the boxed areas in (**b**). (**c**) Expression in the antennal lobes (AL) and subesophageal ganglia (SOG). (**d**) Expression in the mushroom body (MB) lobes (*α*, *β*, and *α*’). (**e**) Expression in the fan-shaped body (FB), ellipsoid body (EB), and noduli (NO) of the central complex. (**f**) Expression in the (**f1**) anterior and (**f2**) posterior mushroom body (MB) peduncles. (**g**) Expression in the protocerebral bridge (PB) of the central complex. (**h**) Expression in the mushroom body (MB) calyx. Neuropils are counter-stained with the nc82 antibody (*α*-Bruchpilot, magenta).

After several failed attempts to generate an antibody that works well for immunohistochemistry, we decided to tag the endogenous Ca-*α*1T with GFP and then visualize its expression pattern in the adult brain. First, we generated a founder line, (*Ca-α1T*^*Founder*, w+^), using end-out homologous recombination to facilitate the versatile generation of a variety of different alleles^15^ (Fig. 2a). In *Ca-α1T*^*Founder*, w+^ flies, an *attP* landing site for *ϕ*C31-mediated DNA integration and a floxed *white*^+^ marker replace ~2 kb of genomic DNA surrounding the first coding exon of Ca-*α*1T. Next, we removed the *white*^+^ marker from the *Ca-α1T*^*Founder*, w+^ line by Cre-mediated recombination to generate *Ca-α1T*^*Founder*, w−^. We then used the *ϕ*C31 integrase to insert into the *attP* landing site of *Ca-α1T*^*Founder*, w−^ an *attB* vector (*pGE-attB*^*GMR*^) containing the deleted genomic region plus an additional GFP coding sequence and linker sequence in-frame after the start codon of Ca-*α*1T. This produced the *GFP::Ca-α1T* line, which expresses an N-terminally GFP-tagged Ca-*α*1T under the control of its own endogenous promoter.

Although *w*^*1118*^ control flies show no fluorescent background (Supplementary Fig. S1), these *GFP::Ca-α1T* flies show GFP fluorescence broadly across the brain (Fig. 2b). *GFP::Ca-α1T* is expressed in well-structured neuropils like the antennal lobes, the mushroom bodies, the central complex (Fig. 2c–h), the optic lobes, as well as in some of the less-structured neuropils. The central complex—comprising the fan-shaped body, ellipsoid body, noduli, and protocerebral bridge—shows the strongest expression with the ventral fan-shaped body and ventral noduli particularly prominent (Fig. 2e,g). In mushroom body neurons, there is far more *GFP::Ca-α1T* in the dendrite-rich calyx of the dorso-posterior brain (Fig. 2h) than the axon-rich lobes of the anterior brain (Fig. 2d). *GFP::Ca-α1T* is also limited to the posterior mushroom body peduncles, which are the fiber tracks that join the posterior calyces with the anterior mushroom body lobes (Fig. 2f). These results suggest strict regulation of the subcellular localization of Ca-*α*1T channels in the brain.

**Figure 3:**
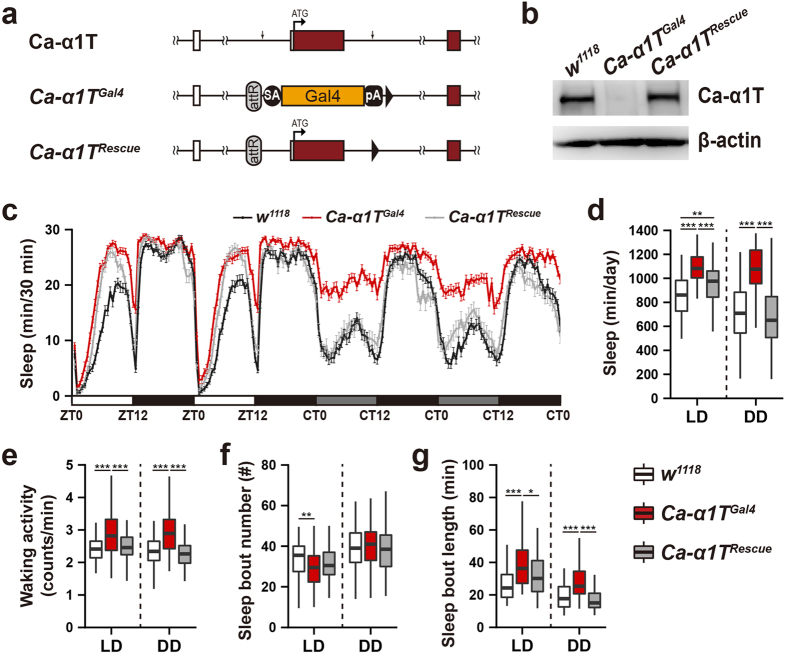
Sleep is increased in *Ca-α1T* mutants. (**a**) *Ca-α1T*, *Ca-α1T*^*Gal4*^, and *Ca-α1T*^*Rescue*^ schematics. Ca-*α*1T coding exons are red. Downward arrows denote the extent of the deleted region. SA, splice acceptor. pA, polyA sequence. (**b**) Western blot analysis of Ca-*α*1T protein levels of fly head lysates. Ca-*α*1T is undetectable in *Ca-α1T*^*Gal4*^ lysates while *Ca-α1T*^*Rescue*^ lysates show levels similar to the *w*^*1118*^ control. *β*-actin was used as a loading control. (**c**) Sleep profiles of *w*^*1118*^ (black, n = 89), *Ca-α1T*^*Gal4*^ (red, n = 92) and *Ca-α1T*^*Rescue*^ (grey, n = 61) over two days of 12 h:12 h light-dark (LD) and two days of continuous dark (DD) conditions. Sleep is plotted in 30 minute intervals. Data are presented as means ± s.e.m. White, black, and grey bars denote light phase, dark phase, and subjective light phase, respectively. ZT, zeitgeber time. CT, circadian time. (**d**) Total daily sleep under LD and DD conditions. (**e**) Waking activity under LD and DD conditions measured as total activity counts divided by waking minutes. (**f**) The number of sleep bouts under LD and DD conditions. (**g**) Average sleep bout length under LD and DD conditions. Boxplot whiskers extend to the highest and lowest values that fall within 1.5× IQR of the upper and lower quartiles. All indications of statistical significance were determined using Welch’s ANOVA followed by the Games-Howell post hoc test. *p < 0.05, **p < 0.01, ***p < 0.001.

We next visualized the projections of Ca-*α*1T-expressing neurons using another knock-in allele, *Ca-α1T*^*Gal4*^. In *Ca-α1T*^*Gal4*^, the first coding exon and flanking introns of Ca-*α*1T are replaced by the Gal4 coding sequence. This puts GAL4 expression under the control of the endogenous Ca-*α*1T promoter (Fig. 3a). Consistent with our results using *GFP::Ca-α1T*, *Ca-α1T*^*Gal4*^ drives the expression of a membrane-tethered mCherry (*UAS-mCD8-ChRFP*) broadly across the brain (Supplementary Fig. S2). The *Ca-α1T*^*Gal4*^ > *mCherry* and *GFP::Ca-α1T* signals are strongly co-localized, including in the central complex and mushroom bodies (Supplementary Fig. S2). This suggests both reagents reflect proper expression from the same endogenous Ca-*α*1T promoter.

### Ca-*α*1T mutants show increased sleep

Since the Gal4 coding sequence inserted into *Ca-α1T*^*Founder*^ to produce the *Ca-α1T*^*Gal4*^ allele included a termination sequence (Fig. 3a), *Ca-α1T*^*Gal4*^ is likely a null allele. As expected, we were unable to detect Ca-*α*1T expression in the fly head lysates from *Ca-α1T*^*Gal4*^ in western blot analyses using polyclonal Ca-*α*1T-specific antisera (Fig. 3b). We did, however, detect strong Ca-*α*1T expression in lysates from *w*^*1118*^ controls and from a *Ca-α1T*^*Rescue*^ allele in which the fragment deleted in both the *Ca-α1T*^*Founder*^ and *Ca-α1T*^*Gal4*^ alleles was re-inserted (Fig. 3a,b). *Ca-α1T*^*Gal4*^ homozygotes are viable and fertile with normal appearance and no obvious movement defects. Two of the mammalian T-type channel subtypes, Ca_v_3.1 and Ca_v_3.3, have been implicated in the generation of the neural oscillations characteristic of NREM sleep^1^,^16^. Flies have a well-established sleep-like state that shares some features with mammalian sleep, but it remains unclear whether flies have a stage akin to mammalian NREM sleep. Still, we hypothesized that Ca-*α*1T-null flies may exhibit sleep defects.

*Ca-α1T*^*Gal4*^ flies show increased total sleep under both 12 h:12 h light-dark (LD) and constant dark (DD) conditions, but this phenotype is particularly prominent during the subjective day under continuous dark (DD) conditions (Fig. 3c,d). The total sleep of *Ca-α1T*^*Rescue*^ flies shows a partial rescue in light-dark (LD) conditions and a full rescue to *w*^*1118*^ levels under continuous darkness (DD) (Fig. 3c,d). Although levels of Ca-*α*1T protein are grossly normal in *Ca-α1T*^*Rescue*^ flies (Fig. 3b), it is possible that the addition of the attR site and the loxP sites in the *Ca-α1T*^*Rescue*^ allele (Fig. 3a) subtly reduce expression of Ca-*α*1T in some small but important neuronal subpopulation preventing a full rescue. By measuring waking locomotor activity, we were able to confirm that the increased sleep of *Ca-α1T*^*Gal4*^ flies is not an artifact of a generalized reduction in movement. In fact, *Ca-α1T*^*Gal4*^ show slightly higher levels of waking activity than their respective controls (Fig. 3e).

Normal fly sleep consists of a number of sleep bouts. We, therefore, asked whether the increased sleep of *Ca-α1T*^*Gal4*^ flies is a result of an increased number of sleep bouts, prolonged bout duration, or both. *Ca-α1T*^*Gal4*^ flies do show reduced sleep bout number under LD conditions, but this phenotype is not rescued in *Ca-α1T*^*Rescue*^ flies (Fig. 3f). Sleep bout length, on the other hand, is increased under both LD and DD conditions and rescued in *Ca-α1T*^*Rescue*^ flies (Fig. 3g).

To confirm that this elevated sleep phenotype is specific to Ca-*α*1T loss-of-function, we generated three independent deletion mutants via imprecise P-element excision. As expected, all three deletion mutants as well as a trans-heterozygous mutants (Δ3/Δ115) show increased sleep, especially in constant darkness (Supplementary Fig. S3). In addition, knockdown of Ca-*α*1T in its own neurons (*Ca-α1T*^*Gal4*^ > *UAS-Ca-α1T-IR*) increases sleep after the third day of continuous darkness (Supplementary Fig. S4). Together, these results implicate Ca-*α*1T as a novel inhibitor of fly sleep.

### Circadian rhythms and sleep homeostasis of Ca-*α*1T mutants

**Figure 4:**
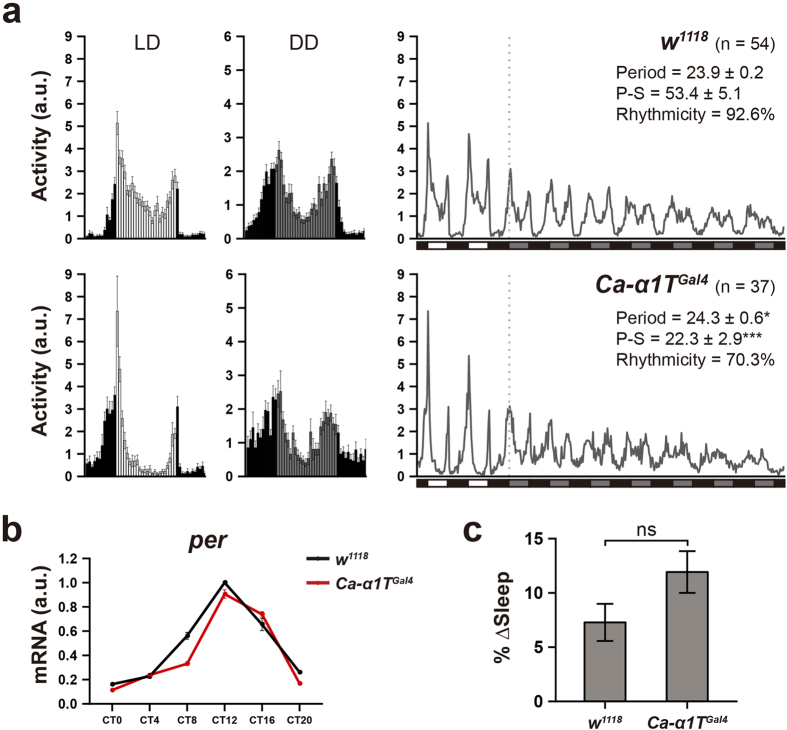
*Ca-α1T*^*Gal4*^ flies show rhythmic locomotion and homeostatic regulation of sleep. (**a**) Average activity profiles from day 2 of the 12 h:12 h light-dark cycles (LD, left), day 2 of continuous darkness (DD, middle), and from throughout the experiment (2 LD + 7 DD, right). In the left and middle panels, data are presented as means ± s.e.m. In the right panel, white, black, and grey bars indicate light phase, dark phase, and subjective light phase, respectively. The dotted line indicates the beginning of constant darkness. The number of flies measured, their rhythmic period, their power of rhythmicity (P-S), and the percentage of rhythmic flies (Rhythmicity) are indicated. a.u., arbitrary unit. The Mann-Whitney U test was used to determine the significance of the period changes (*p < 0.05), while Welch’s t-test was used for rhythmic power (***p < 0.001). (**b**) Transcriptional oscillation of the *period* gene in *Ca-α1T*^*Gal4*^ under DD conditions. Black and red lines denote *w*^*1118*^ and *Ca-α1T*^*Gal4*^, respectively. *rp49* was used for normalization. a.u., arbitrary unit. (**c**) Percentage of lost sleep recovered (% Δ Sleep) over a 12 hr period after 24 hours of mechanically-induced sleep deprivation. *w*^*1118*^ (n = 35) and *Ca-α1T*^*Gal4*^ (n = 33). Statistical significance was determined using the Student’s t-test. ns, not significant. Data are presented as means ± s.e.m.

As in other animals, sleep in *Drosophila* is regulated by the circadian clock, meaning clock mutants generally show altered sleep phenotypes^17^,^18^. We therefore asked whether the increased sleep observed in Ca-*α*1T-null flies can be attributed to a disruption of the circadian clock. After monitoring locomotor activity over seven days of continuous darkness, we found that most *Ca-α1T*^*Gal4*^ flies have a slightly elongated circadian period length (24.3 ± 0.6 vs. 23.9 ± 0.2), a significantly reduced power of rhythmicity (22.3 ± 2.9 vs. 53.4 ± 5.1), and a reduced overall percentage of rhythmic flies (70.3% vs. 92.6%) when compared to *w*^*1118*^ controls (Fig. 4a). This circadian phenotype of *Ca-α1T*^*Gal4*^ flies is unlikely due to problems in the core circadian clock, however, as transcriptional oscillation of *period* is normal (Fig. 4b). This means Ca-*α*1T must act downstream of the core circadian clock to affect rhythmic behaviors, perhaps affecting the firing of important clock-related neurons.

In addition to being controlled by the circadian clock, sleep is also associated with a homeostatic drive proportional to the time an animal spends awake. Thus, we next examined this homeostatic sleep drive in *Ca-α1T*^*Gal4*^ flies by depriving them of sleep for 24 hours and measuring the resulting sleep rebound. *Ca-α1T*^*Gal4*^ flies do recover slightly more of their lost sleep than *w*^*1118*^ controls, but the difference is not statistically significant (Fig. 4c).

### Pan-neuronal knock-down of Ca-*α*1T increases sleep

**Figure 5:**
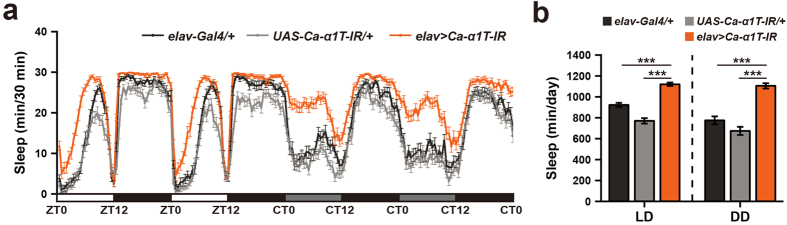
Pan-neuronal Ca-*α*1T knockdown increases sleep. (**a**) Sleep profiles of over two days of 12 h:12 h light-dark cycles (LD) and two days of continuous darkness (DD). Pan-neuronal knockdown of Ca-*α*1T (*elav* > *Ca-α1T-IR*, orange, n = 44) increases sleep beyond that of the heterozygous Gal4 control (*elav-Gal4/*+, black, n = 38) and the heterozygous UAS control (*UAS-Ca-α1T-IR/*+, grey, n = 42). Sleep is plotted in 30 minute intervals. White, black, and grey bars denote light phase, dark phase, and subjective light phase, respectively. ZT, zeitgeber time. CT, circadian time. (**b**) Quantification of average total sleep over two days of light-dark cycles (LD) and two days of continuous darkness (DD). Data are presented as means ± s.e.m. and analyzed via one-way ANOVA followed by the Tukey-HSD post hoc test. ***p < 0.001.

We next asked whether the increased sleep phenotype of *Ca-α1T*^*Gal4*^ flies can be attributed to the function of Ca-*α*1T in the brain. Pan-neuronal knockdown of Ca-*α*1T (*elav-Gal4* > *UAS-Ca-α1T-IR*) increases sleep beyond that of heterozygous controls under both LD and DD conditions (Fig. 5). Using the drug-inducible GeneSwitch-Gal4 technique^19^, we asked whether Ca-*α*1T’s influence on sleep occurs during development or whether it is limited to its expression in the adult brain. Ca-*α*1T knock-down using *elav-GeneSwitch(GS)-Gal4* increases sleep in continuous darkness when compared to non-induced controls (Supplementary Fig. S5). This suggests the sleep phenotype of Ca-*α*1T-null mutants are unlikely due to developmental defects.

**Figure 6:**
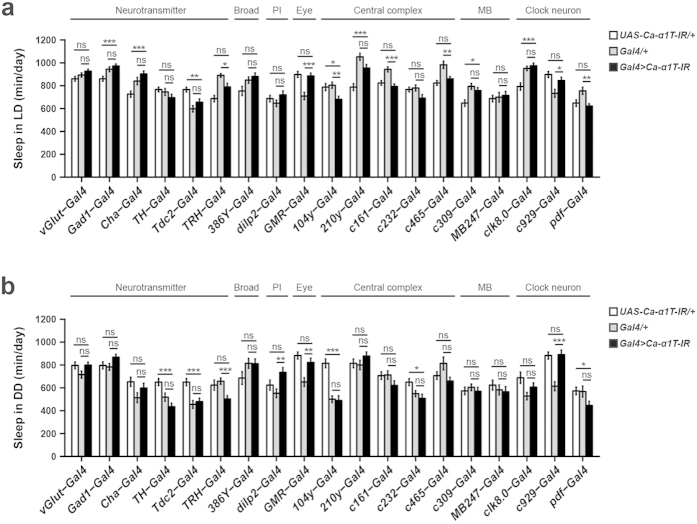
Knockdown of Ca-*α*1T in various neuronal subsets. (**a**) Average total sleep over two days of 12 h:12 h light-dark cycles (LD). (**b**) Average total sleep over two days of continuous darkness (DD). White, grey, and black bars denote *UAS-Ca-α1T-IR/*+, *Gal4/*+ and *Gal4* > *Ca-α1T-IR*, respectively (n = 21–83). PI, pars intercerebralis, MB, mushroom body. Data are presented as means ± s.e.m. Statistical significance was determined using Welch’s ANOVA followed by the Games-Howell post hoc test. ns, not significant. *p < 0.05, **p < 0.01, ***p < 0.001.

Finally, we sought to narrow down the sleep-regulating role of Ca-*α*1T to a specific brain region or circuit. We used a range of neuronal Gal4 drivers that cover known sleep centers to knockdown Ca-*α*1T, but none of them were capable of significantly altering sleep (Fig. 6).

## Discussion

In this study, we cloned the only voltage-gated T-type Ca^2+^ channel from *Drosophila*, Ca-*α*1T. Ca-*α*1T is the largest T-type channel cloned to date, measuring 3205 amino acids^20^. Electrophysiological characterization of Ca-*α*1T in *Xenopus* oocytes showed that Ca-*α*1T has the hallmark properties of a T-type channel: low-threshold activation at around −60 mV, a maximal current output at −20 mV, transient current kinetics elicited by a step-pulse protocol producing a “criss-crossing” pattern, and slow deactivation of tail currents (Fig. 1). These biophysical properties are also consistent with previous studies that implicated Ca-*α*1T in low-voltage-activated (LVA) currents in both the central and peripheral nervous systems of the fly^5^,^21^.

Mammalian genomes contain three T-type Ca^2+^ channel genes (i.e., Ca_v_3.1–3.3), while the fly genome contains only one. We therefore measured Ca-*α*1T for some of the characteristics that distinguish the three mammalian channels. In terms of current kinetics, Ca-*α*1T is more similar to mammalian Ca_v_3.1 and Ca_v_3.2 than Ca_v_3.3, which exhibits considerably slower kinetics. In terms of both its relative permeability to Ba^2+^ over Ca^2+^ and its sensitivity to nickel inhibition, Ca-*α*1T is most similar to Ca_v_3.2^22^,^23^.

The three mammalian T-type Ca^2+^ channels, each with their own distinct biophysical properties, are expressed in largely complementary patterns of neurons throughout the brain, conferring considerable functional diversity. Areas of particularly strong expression include those important for the gating and processing of sensory inputs, motor control, learning and memory, as well as reward circuits^4^. Using a GFP-tagged knock-in allele, we report in this study that Ca-*α*1T is expressed broadly across the adult fly brain in structures reminiscent of the mammalian T-type Ca^2+^ channels. These include sensory neuropils (i.e., the optic and antennal lobes, the antennal mechanosensory and motor centers, the anterior ventrolateral protocerebrum, and the subesophageal zone), motor-associated neuropils (i.e., the central complex), and those associated with learning, memory, and reward (i.e., the mushroom bodies). It is still unclear, however, whether the different isoforms predicted to originate from the *Ca-α1T* locus will have different biophysical properties or different distributions around the brain.

Considering their broad expression, T-type knockout mice appear healthy and subtle mutant phenotypes emerge only upon close inspection. Sleep, in particular, has become a focal point in the search for a physiological function for the T-type channels. Mammalian T-type Ca^2+^ channels may act as sleep stabilizers and may help generate the burst firing necessary for the sleep oscillations of deep NREM sleep. Unfortunately, the three separate mammalian T-type genes all undergo alternative splicing to produce various channel isoforms that each have specific biophysical properties, neuroanatomical and subcellular localizations, and varying abilities to interact with other ion channels. All these variables and more combine to make it difficult if not impossible to define a precise physiological role in sleep for T-type channels as a group. Although Ca_v_3.1 knockout mice lack the delta oscillations characteristic of deep sleep and show reduced total sleep^1^, when the knockout is limited to the rostral midline thalamus, sleep is still reduced, but delta waves are mildly increased^24^. Another more recent study showed that treatment with the T-type-specific channel blocker TTA-A2 enhances sleep and delta rhythms in wild type mice but not Ca_v_3.1/Ca_v_3.3 double knockout mice^25^. In other words, manipulation of T-type channels can both enhance and reduce total sleep and deep delta-wave sleep depending on the experimental context.

Although perhaps an underestimate of the actual complexity of the situation, the subtlety of the phenotypes of the homozygous viable Ca_v_3 mutant mice are often ascribed to functional compensation among the various Ca_v_3.1–3 isoforms^26^. We, therefore, expected that a behavioral investigation of the one and only fly T-type channel, Ca-*α*1T, would uncover less subtle sleep phenotypes. We were thus surprised to find, that despite its broad and relatively strong expression across adult fly brains, Ca-*α*1T-null mutants, like the Ca_v_3.1-null mice, are homozygous viable and lack any overt phenotypes. Upon closer examination, however, we observed that Ca-*α*1T-null mutants sleep more than controls, especially in constant darkness.

The reason for this relative specificity in the sleep phenotype caused by Ca-*α*1T loss-of-function to constant darkness is still unclear. Flies exhibit a burst of activity upon exposure to the early morning light but then sleep through most of the rest of the day. Since control flies show less sleep during subjective daytime under continuous darkness than under the light phase of light-dark conditions (Fig. 3c), it is clear that light exposure can also have sleep-promoting effects. Through a series of imaging experiments, Shang *et al.* reported that although dopamine (DA) is potently wake-promoting, light exposure can suppress this action of DA at least partly by causing the up-regulation of the inhibitory DA receptor D2R in PDF neurons, which are themselves wake-promoting^27^. This modulation of the wake-promoting PDF neurons by light may help explain why the Ca-*α*1T loss-of-function phenotype is biased toward continuous dark conditions if Ca-*α*1T functions downstream of the PDF neurons. It would mean the responsible Ca-*α*1T-positive neurons are also modulated by light.

We were able to replicate the increased sleep phenotype of Ca-*α*1T-null mutants via pan-neuronal knock-down of Ca-*α*1T, but we were unable to further narrow the cause of this phenotype to a more specific neuronal subpopulation. This was in spite of numerous attempts with neuronal Gal4 driver lines ranging from broadly expressed enhancer traps and neurotransmitter Gal4 drivers to much more narrowly expressed neuropeptide drivers. This difficulty suggests Ca-*α*1T may function in novel sleep circuits.

In addition to their sleep phenotype, Ca-*α*1T-null mutants also have a circadian phenotype: an elongated circadian period and a reduction in rhythmic power. It is difficult to say, however, whether these altered circadian parameters are independent of or secondary to the sleep phenotype. Rhythmic power is proportional to the magnitude of the changes in activity level and the regularity with which they occur. Since the increased sleep observed in the Ca-*α*1T-null mutants does reduce the change in overall activity level between subjective day and subjective night, the increased sleep must also cause a reduction in rhythmic power.

The length of time animals spend sleeping is controlled by both the circadian clock and by a homeostatic drive to sleep that is proportional to time spent awake. Thus, most “sleep mutants” described so far have had defects in one or the other—they are either circadian sleep mutants or homeostatic sleep mutants. After 24 hours of mechanically-induced sleep deprivation, we observed that Ca-*α*1T-null mutants re-gain slightly more of their lost sleep than control flies (Fig. 4c), although the increase was not statistically significant. This suggests that, in addition to their circadian phenotype, Ca-*α*1T-null mutants may also have a slightly stronger homeostatic drive to sleep than controls. Although neither the circadian phenotype nor the homeostatic phenotype are particularly strong, together they produce a robust increase in sleep.

The “three channel” compensation hypothesis in mice may yet turn out to be correct, but our results in flies suggest that other factors—isoform-specific differences, differences related to protein–protein interactions, or even something completely unforeseen—may allow mice and flies lacking these broadly expressed and highly conserved ion channels to still function remarkably well. It will be interesting to see whether future studies focused on the technically demanding study of isoform-specific expression patterns and isoform-specific rescues in both mice and flies will clarify how T-type channels can at various times and in various contexts both enhance and reduce sleep.

## Methods

### Fly stocks

Flies were kept on a standard corn meal, corn syrup, yeast, and agar medium at room temperature. *UAS-mCD8-ChRFP* (#27392), *vGlut-Gal4* (#26160), and *Gad1-Gal4* (#47140) were newly obtained from the Bloomington Drosophila Stock Center (Indiana, USA) for these experiments. The UAS-Ca-alpha1T-IR line (#48008) was obtained from the Vienna Drosophila RNAi Center. EP line G1047 was obtained from Genexel. *c465-Gal4* and *210y-Gal4* were gifts from J. Douglas Armstrong^28^. The following stocks were all described previously: *elav-Gal4*^29^, *elav-GS-Gal4*^19^, *Cha-Gal4*^30^, *104y-Gal4*^31^, *c309-Gal4*^32^, *MB247-Gal4*^33^, *pdf-Gal4*^34^, *TH-Gal4*^35^, *GMR-Gal4*^36^, *clk8.0-Gal4*^37^, *dilp2-Gal4*^38^, *Tdc2-Gal4* and *TRH-Gal4*^39^, *c161-Gal4* and *c232-Gal4*^40^, and *c929-Gal4* and *386Y-Gal4*^41^.

### Cloning Ca-*α*1T

We generated a full-length Ca-*α*1T (CG15899) cDNA by piecemeal PCR amplification. Total RNA extracted from adult heads using Trizol reagents (Invitrogen) was reverse transcribed using RevertAid First Strand cDNA Synthesis Kit (Fermentas). Six adjacent DNA fragments that cover the entire Ca-*α*1T cDNA were obtained by PCR amplification. Primer sets were designed based on the FlyBase (FB2011_07) annotation for Ca-*α*1T. Hind III and Xba I sites were inserted at the 5′ end of fragment 1 and 3′ end of fragment 6, respectively. Primer sets: fragment 1 (5′-CGAGATAAGCTTAAAATGCTGCCACAGCCA-3′, 5′-GCATCAGACTACATCGCTGTC-3′), fragment 2 (5′-CTGGACACGCTGCCCATGCTG-3′, 5′-TTCCAGCTCCTCCACTTGCAC-3′), fragment 3 (5′-CAACGGTGGCTCCAACAGTCG-3′, 5′-CCACTGGCGGAAGCTCATGCC-3′), fragment 4 (5′-GCCACGCCTCTCCAAGATCCG-3′, 5′-GACGATAAGAGCGTTTGCACG-3′), fragment 5 (5′-TCTGAAACTAGTCGTGCAAAC-3′, 5′-TGGAAGTACTGGACGGTCTGC-3′), and fragment 6 (5′-AATCCCAGCCTGACCAGCTCG-3′, 5′-TCTAGATTAGTCCATGGAGGATTGGGGTGA-3′). Amplified PCR fragments were sequenced and assembled into pBlueScript II KS (+) using sequential restriction enzyme digests. Clones 2 and 3 contained isoform-specific exons. Of the combinations that were amplified by PCR, we chose to proceed to assembling the RB and RC isoforms. We observed frequent, but inconsistent mutations and instances of A to G RNA editing in fragments 3 and 5. To achieve a final Ca-*α*1T cDNA matching the FlyBase annotation, we reverted one edited site in fragment 3 (5′-AGTTCAGAGC-3′) by site-directed mutagenesis. Since fragment 5 had so many inconsistencies and contained no introns, we used genomic DNA as a template for fragment 5 instead of cDNA. The final assembled full-length cDNAs were cut with HindIII/XbaI and subcloned into pcDNA3-HE3 downstream of the 5′-UTR from the *Xenopus laevis β*-globin gene to improve expression in *Xenopus* oocytes.

### Chemicals and preparation of solutions

Most of the chemicals for electrophysiological recordings were purchased from Sigma-Aldrich (St. Louis, MO, USA). A 100 mM nickel-chloride stock solution was made in deionized water. A series of nickel solutions (in *μ*M: 0.3, 1, 3, 10, 30, 100, 300, 1000, and 3000) were prepared by diluting the stock solution with 10 mM Ba^2+^ recording solution (in mM: 10 BaOH2, 90 NaOH, 1 KOH, 5 HEPES, pH 7.4 adjusted with methanesulfonic acid) before every nickel inhibition experiment.

### Functional expression of T-type channels in *Xenopus* oocytes

Linearized cDNAs encoding rat Ca_v_3.1 or Ca-*α*1T were used as templates for the synthesis of capped cRNAs using T7 RNA polymerase (Ambion, Austin, TX, USA). cRNA concentrations were estimated based on spectrophotometric optical density measurements at 260 nm. Oocyte preparation from female *Xenopus laevis* and injection of cRNAs was performed as previously reported^22^. GenBank accession numbers: rat Ca_v_3.1 (*α*_1_G), AF027984^6^; Ca-*α*1T C isoform, NP001096889.

### Electrophysiology

Ba^2+^ (or Ca^2+^) currents through T-type channels expressed in oocytes were measured at room temperature 4–5 days after cRNA injection using a two-electrode voltage-clamp amplifier (OC-725C, Warner Instruments, Hamden, CT, USA). Microelectrodes were pulled from capillaries (G100TF-4, Warner Instruments, Hamden, CT, USA) using a pipette puller and filled with 3 M KCl. All electrodes used measured 0.5–1.1 MΩ of resistance. The 10 mM Ba^2+^ (or Ca^2+^) recording solution contained: 10 mM Ba(OH)_2_ (or Ca(OH)_2_), 90 mM NaOH, 1 mM KOH, 5 mM HEPES (pH 7.4, adjusted with methanesulfonic acid). To remove any contamination from Ca^2+^-activated chloride currents, we injected the oocytes with 50 nL of 50 mM BAPTA (1,2-bis[o-aminophenoxy] ethane -N,N,N’,N’-tetraacetic acid) 30–60 min prior to recording. This was especially important while recording Ca^2+^ currents. Currents were sampled at 5 kHz and low pass filtered at 1 kHz using the pClamp system (Digidata 1320A and pClamp 8; Axon instruments, Foster City, CA, USA) unless otherwise noted.

We used whole cell patch clamp recordings from HEK-293 cells transiently transfected with Ca-*α*1T to measure tail currents. These recordings were obtained at room temperature using an Axopatch 200A amplifier connected to a computer through a Digidata 1300 A/D converter and controlled with the pCLAMP 9.2 software. Tail currents were recorded in a 10 mM Ba^2+^ solution containing the following: 140 mM TEACl, 2.5 mM CsCl, 10 mM BaCl_2_, 1 mM MgCl_2_, 10 mM glucose, and 10 mM HEPES (pH 7.3, adjusted with TEAOH). The pipette solution contained the following: 130 mM CsCl, 10 mM EGTA, 5 mM MgATP, 1 mM NaGTP, and 10 mM HEPES (pH 7.4, adjusted with CsOH). Recording pipettes were prepared from TW-150-3 capillaries (World Precision Instruments, Inc., Sarasota, FL). The pipette resistance was 2.0 ~ 3.0 MΩ. Access resistance was compensated by 70–80% using the compensation circuit and series resistance prediction. Tail current data were filtered at 10 kHz and digitized at 20 kHz. Peak currents and exponential fits were analyzed using the Clampfit software package (Axon instruments, Foster City, CA, USA). The activation and inactivation time constants for the T-type currents elicited by step pulse protocols were estimated by fitting individual current traces with double exponential functions: 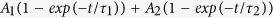 where *A*_1_ and *A*_2_ are the coefficients for the activation and inactivation exponentials, *t* is time, and *τ*_1_ and *τ*_2_ are the activation and inactivation time constants, respectively. The smooth curves for channel activation and steady-state inactivation were obtained by fitting the data with a Boltzmann equation: 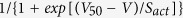, where *V*_50_ is the potential for half-maximal activation and *S*_*act*_ is the slope conductance. Dose-response curves for Ni^2+^ inhibition of T-type channel currents were derived by fitting the data using a Hill equation: 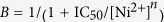, where *B* is the normalized block, IC_50_ is the concentration of Ni^2+^ giving half maximal blockade, and *n* is the Hill coefficient.

### Generation of knock-in alleles

5′ and 3′ homologous arms surrounding the *Ca-α1T* locus were PCR-amplified using *w*^*1118*^ genomic DNA with the following primers: 5′-CGAGATGAATTCTAGCCTCATCAACTGAGC-3′, 5′-CGAGATGCGGCCGCGAGCAAGCACTAATAGCA-3′, 5′-GAGATACTAGTCATGCTACAATGTCAGCA-3′, 5′-CGAGATCTCGAGGGCCACGTATAGGGATGC-3′. The homologous arms were then inserted into the *pGX-attP* vector (DGRC #1293). P{Donor} flies were generated by P-element based transgenesis of *pGX-attP* containing the homologous arms into the *w*^*1118*^ genetic background (Genetic Services, Inc., US) and crossed to Flp I-Sce I flies for homologous recombination. Candidates for proper targeting (i.e., flies with red or mosaic eyes) were selected and verified by PCR. The *white* marker was removed from a verified strain via Cre-mediated recombination. The resulting *white*^−^ line was used as a founder (*Ca-α1T*^*Founder, w*−^) for site-specific DNA integration. *Ca-α1T*^*Gal4*^, *Ca-α1T*^*Rescue*^ and *GFP::Ca-α1T* lines were generated by *ϕ*C31 integrase-mediated site-specific integration. The Gal4 insert (i.e., splice acceptor-Gal4 CDS-poly A) was amplified from the *pBS-KS-attB1-2-GT-SA-GAL4-Hsp70pA* vector (DGRC #1325) with the following primers: 5′-CGTACTCCACGAATTTCTAGAAGTCGATCCAACAT-3′ and 5′-ACCGGCGCGCCTCGACTCTAGAACTAGTGGATCTA-3′. The resulting amplified DNA fragment was sequenced and inserted into the *pGE-attB*^*GMR*^ vector (DGRC #1295) using the EZ-FusionTM cloning kit (Enzynomics, South Korea). The Rescue insert was PCR amplified from *w*^*1118*^ genomic DNA with the following primers: 5′-GCAGAATTCAATCGATTCCATAGATCCGC-3′ and 5′-GCACTCGAGAATTTTGCAACAGGCAGCTA-3′. The resulting fragment was inserted into the EcoR I/Xho I site of the *pGE-attB*^*GMR*^ vector. The GFP insert along with a (Gly-Gly-Ser)x4 linker was amplified from the *pBS-KS-attB1-2-PT-SA-SD-1-EGFP-FIAsH-StrepII-TEV-3xFlag* vector (DGRC #1306) with the following primers: 5′-GCACCCCAGAAAATGGTGTCCAAGGGCGAGGAGCT-3′ and 5′-CGCTGGCTGTGGCAGGGAACCTCCGCTTCCACCGC-3′. The resulting fragment was inserted downstream of the ATG start site in the Rescue construct by inverse PCR (5′-CTGCCACAGCCAGCGGCAGCG-3′, 5′-CATTTTCTGGGGTGCCAACTA-3′) using the 5X In-Fusion HD Enzyme Premix (Clontech). *pGE-attB*^*GMR*^ vectors containing the Gal4, Rescue, and GFP-tagging constructs were injected into *Ca-α1T*^*Founder, w*−^ embryos (Rainbow Transgenic Flies, Inc., US) for *ϕ*C31-mediated site-specific integration into the *attP* landing site in the Ca-*α*1T locus. The *Ca-α1T*^*Gal4*^ and *Ca-α1T*^*Rescue*^ lines were backcrossed to *w*^*1118*^ for more than 8 generations and their white-markers were removed before behavioral analysis.

### Generation of deletion mutants of Ca-*α*1T using imprecise P-element excision

EP line G1047 from the Genexel collection was crossed to the transposase line (*Dr[1]/TMS,Sb,P[*Δ*2*–*3]*). Mosaic-eyed progeny were collected and crossed to an X chromosome balancer (*phl12/FM6*) to obtain candidate excision lines. These candidates were then verified by PCR and backcrossed to *w*^*1118*^ for more than 8 generations before continuing to the sleep analyses.

### Antibody generation and western blotting

A polyclonal antisera against Ca-*α*1T was generated using antigen derived from the 302 C-terminal amino acids of Ca-*α*1T (cloning primers: 5′-GAATTCCAAATTAATCCAATCCGTA-3′, 5′-GCGGCCGCTTAGTCCATGGAGGATT-3′). His-tagged antigen was expressed in *E. coli*, purified and injected into rabbits to generate an immune response (YoungIn Frontier, South Korea). Western blot analyses were performed according to standard protocols using rabbit antisera obtained after the third Ca-*α*1T antigen boost. *β*-Actin-specific antibodies (Santa Cruz Biotechnology, sc-47778) were used for the loading control.

### Immunohistochemistry

Adult female fly brains were dissected in PBS and fixed with 4% PFA for 30 minutes at room temperature. Fixed brain samples were washed with PAT3 solution (0.5% TritonX-100, 0.5% BSA in PBS) for 15 min 3 times and incubated in 5% normal goat serum for 2–3 hours at room temperature. After blocking, samples were incubated with primary antibodies diluted in 5% normal goat serum overnight at 4 °C. The samples were then washed with PAT3 for 1 hour 2 times at room temperature and incubated with secondary antibodies diluted in 5% normal goat serum overnight at 4 °C. After washing off the secondary antibodies with PAT3 for 1 hour 2 times, the brain samples were mounted in Vectashield (H-1000, Vector Laboratories, Inc.) and visualized on a LSM-780 confocal microscope (Zeiss, Germany). Primary antibodies: rabbit anti-GFP (1:500, A11122, Invitrogen); anti-bruchpilot monoclonal (1:50, nc82, DSHB). Secondary antibodies: goat anti-rabbit Alexa 488 (1:300, A11008, Invitrogen); goat anti-mouse Alexa 568 (1:300, A11031, Invitrogen).

### Sleep and locomotor behavior analysis

Fly sleep and locomotor behavior was measured with the *Drosophila* Activity Monitoring system (Trikinetics). For sleep analysis, 3–4 day-old female flies were placed individually into 65 mm X 5 mm glass tubes with one end filled with 2% agar/5% sucrose food and the other end plugged with cotton. We defined periods of activity as periods with a beam break frequency higher than 1 per minute and periods of sleep as periods during which no beam break occurred for at least 5 consecutive minutes^42^. After one day of habituation in an incubator (25 °C, 60% humidity), we used the “Counting Macro” software^43^ to measure sleep over the course of 4 days—2 days of 12 hr:12 hr light-dark conditions and 2 days of continuous darkness. For experiments using the GeneSwitch technique, flies were maintained on normal food containing 500 *μ*M RU486 (M8046, Sigma-Aldrich) dissolved in ethanol (1%) for two days prior to the experiment. Control flies were maintained on normal food containing only ethanol (1%). For the GeneSwitch experiments, flies were placed in 2% agar/5% sucrose food with or without 500 *μ*M RU486. For sleep deprivation, activity monitors with 3–5 day-old female flies were placed in a Sleep Nullifying Apparatus (SNAP)^44^ designed to rotate and give a swift mechanical stimulus twice per minute. After three days under 12 hr:12 hr light-dark conditions, flies were sleep-deprived for 24 hr and allowed to recover 12 hr. The percentage of lost sleep recovered (% Δ Sleep) was calculated by subtracting the baseline sleep (i.e., sleep during the light phase immediately before the deprivation day) from the amount of sleep during the recovery period and then dividing by sleep lost. We confirmed that each genotype lost 90% of their baseline sleep during the deprivation period and we included only flies with ≥70% sleep lost in the following calculations. For the circadian locomotor analyses, we measured the activity of 1–3 day-old male flies in 30 minute bins and analyzed the data using ClockLab (Actimetrics) and the Counting Macro^45^. Significance level for the *χ*^2^ periodogram was set to *α* = 0.05. Flies with a power of significance (P-S) ≥10 were considered rhythmic.

### Quantitative real-time PCR

Male adult flies were maintained for 2 days under 12 h:12 h light-dark conditions and 2 days of continuous darkness. 120–150 fly heads were collected for each time point (CT0-CT20) during the second day of continuous darkness. Total RNA was extracted using Trizol (Invitrogen) and reverse transcribed using the RevertAid First Strand cDNA Synthesis Kit (Thermo Scientific). Quantitative real-time PCR was performed using the TOPreal qPCR 2× premix (RT500M, Enzynomics, South Korea). Expression levels of *period* were normalized to the level of *rp49*. Primers: *per* (5′-GACCGAATCCCTGCTCAATA-3′, 5′-GTGTCATTGGCGGACTTCTT-3′); *rp49* (5′-ATGACCATCCGCCCAGCATA-3′, 5′-GAGAACGCAGGCGACCGTTG-3′).

### Statistical analysis

All statistical analysis was performed using R (version 3.2.0)^46^ except for the analysis of the electrophysiological recordings, which was performed in GraphPad (San Diego, CA, USA). For comparisons between two genotypes, we used the Student’s t-test or Welch’s t-test as described in the figure legends. The Mann-Whitney U test was substituted when the data did not follow a normal distribution. For comparisons among three or more genotypes, we used the one-way ANOVA followed by the Tukey HSD post hoc test. When the data had unequal variance, we used Welch’s one-way ANOVA followed by the Games-Howell post hoc test. We used the “car”^47^ and “userfriendlyscience”^48^ R packages to perform Levene’s test for homogeneity of variance and the Games-Howell post hoc test, respectively.

## Additional Information

**How to cite this article**: Jeong, K. *et al.* Ca-α1T, a fly T-type Ca^2+^ channel, negatively modulates sleep. *Sci. Rep.* **5**, 17893; doi: 10.1038/srep17893 (2015).

## Supplementary Material

Supplementary Information

## Figures and Tables

**Table 1:** Comparison of the biophysical properties of Ca-*α*1T and mammalian T-type calcium channels.

	*Ca-α1T*	*Rat Ca*_*v*_*3.1*	*Ca*_*v*_*3.1*	*Ca*_*v*_*3.2*	*Ca*_*v*_*3.3*
Activation					
V_50_ (mV)	−43.3 ± 1.6 (11)	−38.9 ± 1.2 (14)	−**40.0 ± 0.8**^49^	−36.2 ± 0.6^49^	−29.1 ± 1.3^49^
*k* (mV)	7.7 ± 1.3 (11)	6.4 ± 0.9 (14)	NA	NA	NA
Inactivation					
V_50_ (mV)	−58.0 ± 0.7 (5)	−61.3 ± 0.7 (15)	−64.9 ± 0.7^49^	−62.0 ± 0.9^49^	−**55.6 ± 0.9**^49^
Current kinetics (−20 mV)					
*τ*_act_ (ms)	2.2 ± 0.2 (10)	1.1 ± 0.2 (5)	**1.4 ± 0.1**^50^	3.2 ± 0.1^51^	22.8 ± 1.5^50^
*τ*_inact_ (ms)	23.4 ± 1.4 (6)	9.7 ± 0.9 (5)	7.2 ± 0.4^50^	**11.6 ± 0.2**^51^	97.0 ± 3.8^50^
Deactivation kinetics (−100 mV)					
*τ*_deact_ (ms)	0.9 ± 0.1 (6)^a^	NA	2.6 ± 0.2^52^^ ^a,b	3.6 ± 0.4^52^^ ^a,b	**1.12 ± 0.1**^52^^** **^a,b
Conductance					
I_Ca_/I_Ba_	0.7 (6)	1.6 (3)	1.5^13^	0.8^13^	**0.7**^13^
Pharmacology					
Ni^2+^ (IC_50_ *μ*M)	5.1 (6–12)	276.5 (5–6)	167 ± 15^14^^ ^c	**5.7 ± 0.3**^14^^** **^c	87 ± 7^14^^ ^c

V_50_, potential of half maximal activation or inactivation. *k*, slope factor from the Boltzmann equation. Number of oocytes is in parentheses. The measurements reported in the first two columns labeled Ca-*α*1T and Rat Ca_v_3.1 were measured in this study, while the final three columns are from published reports. Bold-faced entries in the final three columns denote the mammalian T-type channel properties that most closely match the measured properties of Ca-*α*1T. NA, not available.

^a^Recorded in HEK-293 cells.

^b^2 mM Ca^2+^ instead of 10 mM Ba^2+^ as charge carrier.

^c^−30 mV as repolarizing potential.

## References

[b1] Lee J., Kim D. & Shin H.-S. Lack of delta waves and sleep disturbances during non-rapid eye movement sleep in mice lacking alpha1G-subunit of T-type calcium channels. Proc. Natl. Acad. Sci. USA 101, 18195–18199 (2004). 15601764 10.1073/pnas.0408089101 PMC539778

[b2] Steriade M., Dossi R. C. & Nuñez A. Network modulation of a slow intrinsic oscillation of cat thalamocortical neurons implicated in sleep delta waves: cortically induced synchronization and brainstem cholinergic suppression. J. Neurosci. 11, 3200–3217 (1991). 1941080 10.1523/JNEUROSCI.11-10-03200.1991 PMC6575431

[b3] Dossi R. C., Nuñez A. & Steriade M. Electrophysiology of a slow (0.5-4 hz) intrinsic oscillation of cat thalamocortical neurones *in vivo*. J. Physiol. 447, 215–234 (1992). 1593448 10.1113/jphysiol.1992.sp018999 PMC1176033

[b4] Talley E. M. *et al.* Differential distribution of three members of a gene family encoding low voltage-activated (T-type) calcium channels. J. Neurosci. 19, 1895–1911 (1999). 10066243 10.1523/JNEUROSCI.19-06-01895.1999 PMC6782581

[b5] Ryglewski S., Lance K., Levine R. B. & Duch C. Ca(v)2 channels mediate low and high voltage-activated calcium currents in Drosophila motoneurons. J. Physiol. 590, 809–825 (2012). 22183725 10.1113/jphysiol.2011.222836 PMC3381312

[b6] Perez-Reyes E. *et al.* Molecular characterization of a neuronal low-voltage-activated T-type calcium channel. Nature 391, 896–900 (1998). 9495342 10.1038/36110

[b7] Carbone E. & Lux H. A low voltage-activated, fully inactivating Ca channel in vertebrate sensory neurones. Nature 310, 501–502 (1984). 6087159 10.1038/310501a0

[b8] Cribbs L. L. *et al.* Cloning and characterization of alpha1H from human heart, a member of the T-type Ca^2+^ channel gene family. Circ. Res. 83, 103–109 (1998). 9670923 10.1161/01.res.83.1.103

[b9] Lee J.-H. *et al.* Cloning and expression of a novel member of the low voltage-activated T-type calcium channel family. J. Neurosci. 19, 1912–1921 (1999). 10066244 10.1523/JNEUROSCI.19-06-01912.1999 PMC6782566

[b10] Klöckner U. *et al.* Comparison of the Ca^2+^ currents induced by expression of three cloned *α*1 subunits, *α*1G, *α*1H and *α*1I, of low-voltage-activated T-type Ca^2+^ channels. Eur. J. Neurosci. 11, 4171–4178 (1999). 10594642 10.1046/j.1460-9568.1999.00849.x

[b11] Matteson D. & Armstrong C. Properties of two types of calcium channels in clonal pituitary cells. J. Gen. Physiol. 87, 161–82 (1986). 2419479 10.1085/jgp.87.1.161 PMC2217130

[b12] McRory J. E. *et al.* Molecular and functional characterization of a family of rat brain T-type calcium channels. J. Biol. Chem. 276, 3999–4011 (2001). 11073957 10.1074/jbc.M008215200

[b13] Shcheglovitov A., Kostyuk P. & Shuba Y. Selectivity signatures of three isoforms of recombinant T-type Ca^2+^ channels. Biochim. Biophys. Acta, Biomembr. 1768, 1406–1419 (2007). 10.1016/j.bbamem.2007.02.017 17400181

[b14] Lee J.-H., Gomora J. C., Cribbs L. L. & Perez-Reyes E. Nickel block of three cloned T-type calcium channels: low concentrations selectively block *α*1H. Biophys. J. 77, 3034–3042 (1999). 10585925 10.1016/S0006-3495(99)77134-1 PMC1300574

[b15] Huang J., Zhou W., Dong W., Watson A. M. & Hong Y. Directed, efficient, and versatile modifications of the Drosophila genome by genomic engineering. Proc. Natl. Acad. Sci. USA 106, 8284–8289 (2009). 19429710 10.1073/pnas.0900641106 PMC2688891

[b16] Astori S. *et al.* The CaV3.3 calcium channel is the major sleep spindle pacemaker in thalamus. Proc. Natl. Acad. Sci. USA 108, 13823–13828 (2011). 21808016 10.1073/pnas.1105115108 PMC3158184

[b17] Hendricks J. *et al.* Gender dimorphism in the role of cycle (BMAL1) in rest, rest regulation, and longevity in Drosophila melanogaster. J. Biol. Rhythms. 18, 12–25 (2003). 12568241 10.1177/0748730402239673

[b18] Parisky K. *et al.* PDF cells are a GABA-responsive wake-promoting component of the Drosophila sleep circuit. Neuron 60, 672–82 (2008). 19038223 10.1016/j.neuron.2008.10.042 PMC2734413

[b19] Osterwalder T., Yoon K. S., White B. H. & Keshishian H. A conditional tissue-specific transgene expression system using inducible GAL4. Proc. Natl. Acad. Sci. USA 98, 12596–12601 (2001). 11675495 10.1073/pnas.221303298 PMC60099

[b20] Senatore A. & Spafford J. D. Transient and big are key features of an invertebrate T-type channel (LCa_v_3) from the central nervous system of Lymnaea stagnalis. J. Biol. Chem. 285, 7447–7458 (2010). 20056611 10.1074/jbc.M109.090753 PMC2844193

[b21] Iniguez J., Schutte S. S. & O’Dowd D. K. Cav3-type *α*1T calcium channels mediate transient calcium currents that regulate repetitive firing in Drosophila antennal lobe PNs. J. Neurophysiol. 110, 1490–1496 (2013). 23864373 10.1152/jn.00368.2013 PMC4042424

[b22] Kang H. *et al.* A molecular determinant of nickel inhibition in Cav3.2 T-type calcium channels. J. Biol. Chem. 281, 4823–30 (2006). 16377633 10.1074/jbc.M510197200

[b23] Park H. *et al.* Asp residues of the Glu-Glu-Asp-Asp pore filter contribute to ion permeation and selectivity of the Ca(v)3.2 T-type channel. Cell Calcium 54, 226–35 (2013). 23849427 10.1016/j.ceca.2013.06.006

[b24] Anderson M. P. *et al.* Thalamic Cav3.1 t-type Ca^2+^ channel plays a crucial role in stabilizing sleep. Proc. Natl. Acad. Sci. USA 102, 1743–1748 (2005). 15677322 10.1073/pnas.0409644102 PMC547889

[b25] Kraus R. L. *et al.* *In vitro* characterization of T-type calcium channel antagonist TTA-A2 and *in vivo* effects on arousal in mice. J. Pharmacol. Exp. Ther. 335, 409–417 (2010). 20682849 10.1124/jpet.110.171058

[b26] Senatore A., Zhorov B. S. & Spafford J. D. Cav3 T-type calcium channels. WIREs Membr. Transp. Signal. 1, 467–491 (2012).

[b27] Shang Y. *et al.* Imaging analysis of clock neurons reveals light buffers the wake-promoting effect of dopamine. Nat. Neurosci. 14, 889–895 (2011). 21685918 10.1038/nn.2860 PMC3424274

[b28] Young J. M. & Armstrong J. D. Structure of the adult central complex in Drosophila: organization of distinct neuronal subsets. J. Comp. Neurol. 518, 1500–1524 (2010). 20187142 10.1002/cne.22284

[b29] Lin D. M. & Goodman C. S. Ectopic and increased expression of Fasciclin II alters motoneuron growth cone guidance. Neuron 13, 507–523 (1994). 7917288 10.1016/0896-6273(94)90022-1

[b30] Kitamoto T. Conditional modification of behavior in Drosophila by targeted expression of a temperature-sensitive shibire allele in defined neurons. J. Neurobiol. 47, 81–92 (2001). 11291099 10.1002/neu.1018

[b31] Sakai T. & Kitamoto T. Differential roles of two major brain structures, mushroom bodies and central complex, for Drosophila male courtship behavior. J. Neurobiol. 66, 821–834 (2006). 16673386 10.1002/neu.20262

[b32] Connolly J. B. *et al.* Associative learning disrupted by impaired Gs signaling in *Drosophila* mushroom bodies. Science 274, 2104–2107 (1996). 8953046 10.1126/science.274.5295.2104

[b33] Zars T., Fischer M., Schulz R. & Heisenberg M. Localization of a short-term memory in *Drosophila*. Science 288, 672–675 (2000). 10784450 10.1126/science.288.5466.672

[b34] Renn S. C., Park J. H., Rosbash M., Hall J. C. & Taghert P. H. A pdf neuropeptide gene mutation and ablation of PDF neurons each cause severe abnormalities of behavioral circadian rhythms in *Drosophila*. Cell 99, 791–802 (1999). 10619432 10.1016/s0092-8674(00)81676-1

[b35] Friggi-Grelin F. *et al.* Targeted gene expression in *Drosophila* dopaminergic cells using regulatory sequences from tyrosine hydroxylase. J. Neurobiol. 54, 618–627 (2003). 12555273 10.1002/neu.10185

[b36] Freeman M. Reiterative use of the EGF receptor triggers differentiation of all cell types in the Drosophila eye. Cell 87, 651–660 (1996). 8929534 10.1016/s0092-8674(00)81385-9

[b37] Glossop N. R. *et al.* VRILLE feeds back to control circadian transcription of Clock in the *Drosophila* circadian oscillator. Neuron 37, 249–261 (2003). 12546820 10.1016/s0896-6273(03)00002-3

[b38] Rulifson E. J., Kim S. K. & Nusse R. Ablation of insulin-producing neurons in flies: growth and diabetic phenotypes. Science 296, 1118–1120 (2002). 12004130 10.1126/science.1070058

[b39] Alekseyenko O. V., Lee C. & Kravitz E. A. Targeted manipulation of serotonergic neurotransmission affects the escalation of aggression in adult male *Drosophila melanogaster*. PLoS One 5, e10806 (2010). 20520823 10.1371/journal.pone.0010806 PMC2875409

[b40] Renn S. C. *et al.* Genetic analysis of the *Drosophila* ellipsoid body neuropil: organization and development of the central complex. J. Neurobiol. 41, 189–207 (1999). 10512977

[b41] Taghert P. H. *et al.* Multiple amidated neuropeptides are required for normal circadian locomotor rhythms in Drosophila. J. Neurosci. 21, 6673–6686 (2001). 11517257 10.1523/JNEUROSCI.21-17-06673.2001 PMC6763108

[b42] Shaw P. J., Cirelli C., Greenspan R. J. & Tononi G. Correlates of sleep and waking in Drosophila melanogaster. Science 287, 1834–1837 (2000). 10710313 10.1126/science.287.5459.1834

[b43] Pfeiffenberger C., Lear B., Keegan K. & Allada R. Processing sleep data created with the Drosophila Activity Monitoring (DAM) System. Cold Spring Harb. Protoc. 2010, pdb.prot5520 (2010). 10.1101/pdb.prot5520 21041393

[b44] Shaw P. J., Tononi G., Greenspan R. J. & Robinson D. F. Stress response genes protect against lethal effects of sleep deprivation in drosophila. Nature 417, 287–291 (2002). 12015603 10.1038/417287a

[b45] Pfeiffenberger C., Lear B., Keegan K. & Allada R. Processing circadian data collected from the Drosophila Activity Monitoring (DAM) System. Cold Spring Harb. Protoc. 2010, pdb.prot5519 (2010). 10.1101/pdb.prot5519 21041392

[b46] R Core Team. R: A Language and Environment for Statistical Computing. R Foundation for Statistical Computing, Vienna, Austria (2015).

[b47] Fox J. & Weisberg S. An R Companion to Applied Regression (Sage, Thousand Oaks CA, 2011), second edn.

[b48] Peters G.-J. *userfriendlyscience: Quantitative analysis made accessible* (2015). R package version 0.3-0.

[b49] Park J.-Y. *et al.* Activation of protein kinase C augments T-type Ca^2+^ channel activity without changing channel surface density. J. Physiol. 577, 513–523 (2006). 17008378 10.1113/jphysiol.2006.117440 PMC1890444

[b50] Park J., Kang H., Jeong S. & Lee J. Multiple structural elements contribute to the slow kinetics of the Cav3.3 T-type channel. J. Biol. Chem. 279, 21707–13 (2004). 15016809 10.1074/jbc.M400684200

[b51] Demers-Giroux P., Bourdin B., Sauvé R. & Parent L. Cooperative activation of the T-type Ca_v_3.2 channel: interaction between Domains II and III. J. Biol. Chem. 288, 29281–93 (2013). 23970551 10.1074/jbc.M113.500975 PMC3795230

[b52] Chemin J. *et al.* Specific contribution of human T-type calcium channel isotypes (alpha(1G), alpha(1H) and alpha(1I)) to neuronal excitability. J. Physiol. 540, 3–14 (2002). 11927664 10.1113/jphysiol.2001.013269 PMC2290209

