# Supplementary material for: Ca-α1T, a fly T-type Ca2+ channel, negatively modulates sleep
Source: Sci Rep. 2015 Dec 9;5:17893. doi: 10.1038/srep17893 (PMC4673464; doi:10.1038/srep17893)
Supplement: Supplementary Information [file srep17893-s1.pdf]

Ca-α1T, a fly T-type Ca^{2+} channel, negatively modulates sleep

Kyunghwa Jeong[1], Soyoung Lee[2], Haengsoo Seo[2], Yangkyun Oh[1], Donghoon Jang[1], Joonho Choe[1], Daesoo Kim[1], Jung-Ha Lee[2,+], and Walton D. Jones[1,*]

[1]KAIST, Department of Biological Sciences, Daejeon, 305-701, Republic of Korea
[2]Sogang University, Department of Life Sciences, Seoul, 121-742, Republic of Korea
[+]jhleem@sogang.ac.kr
[*]waltonjones@kaist.edu

Supplementary Figures

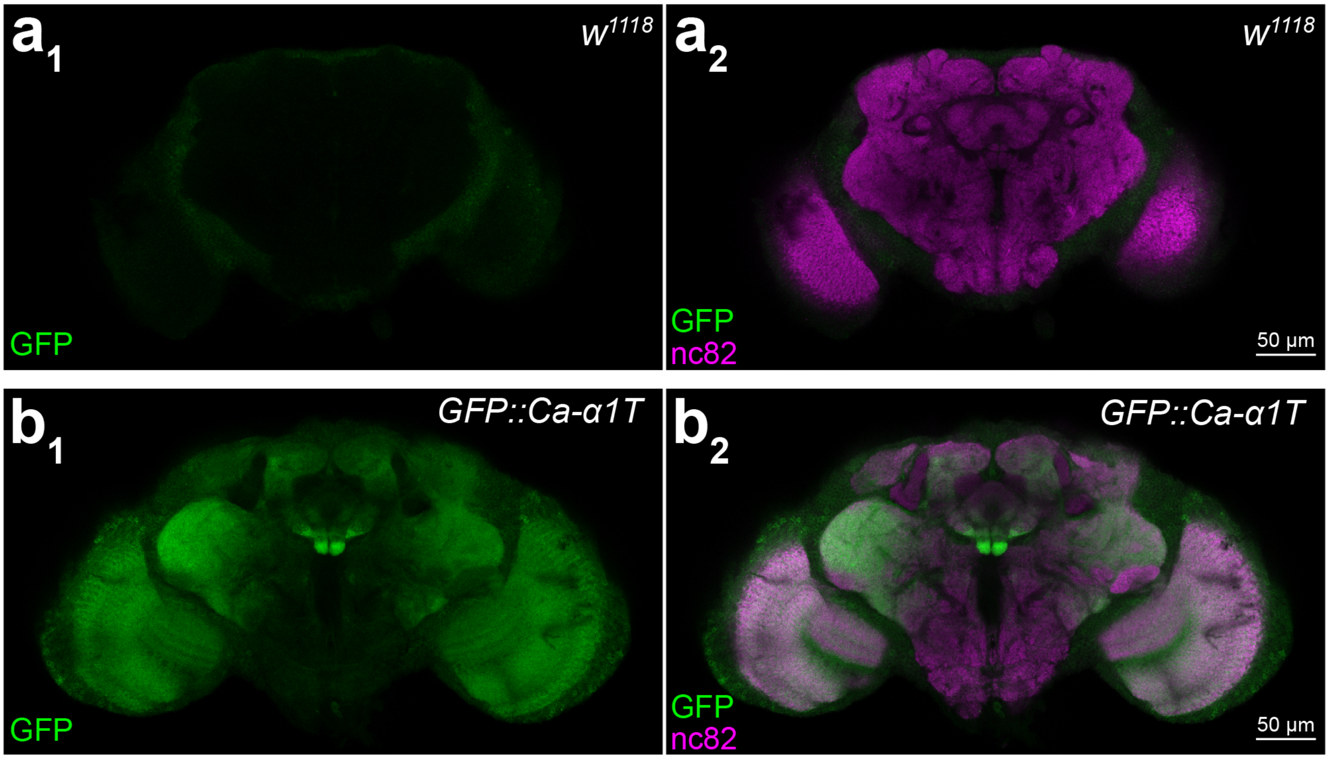

Supplementary Figure S1. Staining of GFP in *w*^{1118} control and *GFP::Ca-*α*1T*
(a) (a1) Adult brain of *w*^{1118} control flies stained with the GFP antibody (green). **(a2)** Co-staining with the nc82 antibody (magenta) for visualizing neuropil structures. **(b) (b1)** Adult brain of *GFP::Ca-*α*1T* stained with the GFP antibody (green). **(b2)** Co-staining with the nc82 antibody (magenta) for visualizing neuropil structures.

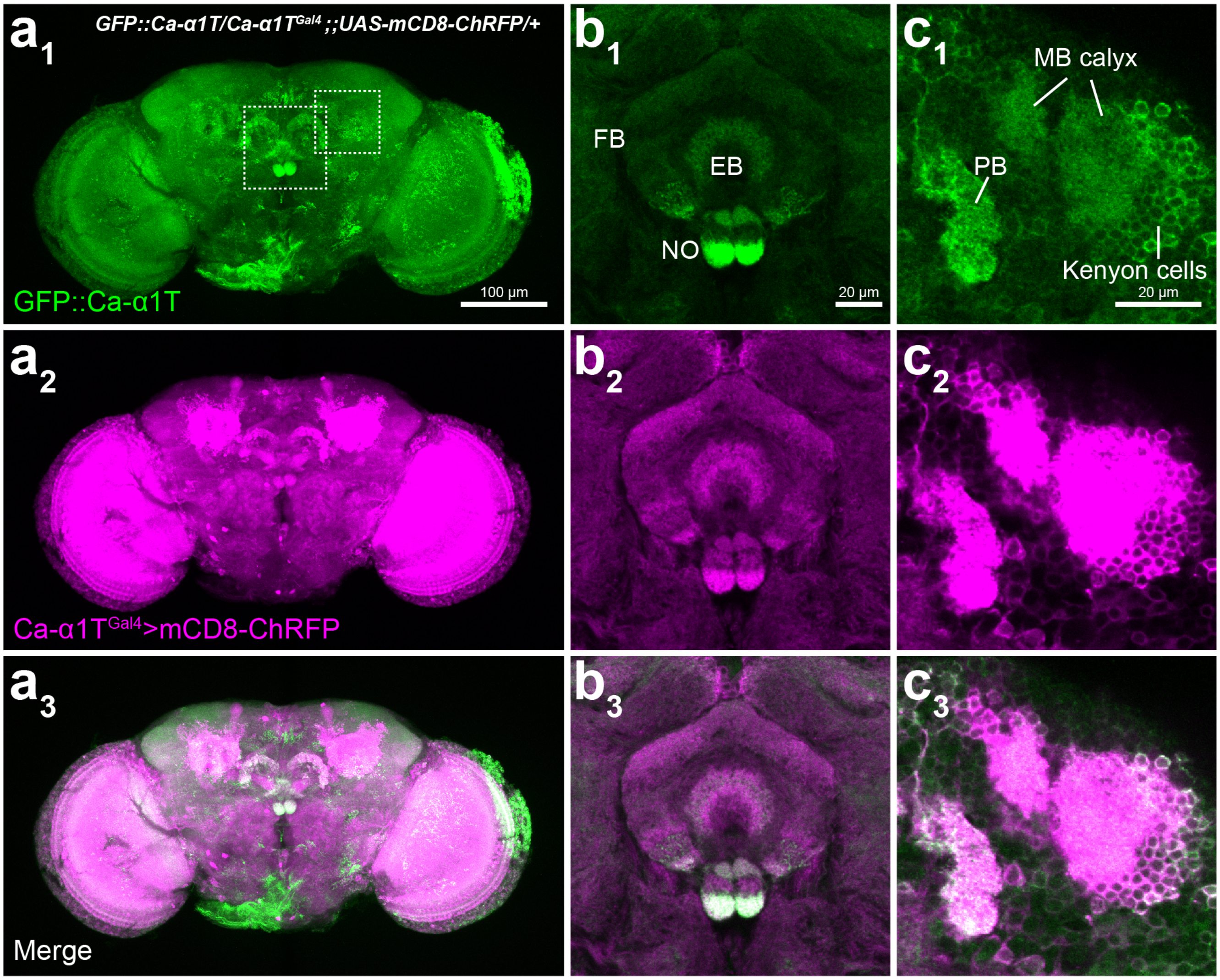

Supplementary Figure S2. ***GFP::Ca-α1T* and *Ca-α1T^{Gal4}* label the same neurons.**
(a) GFP::Ca-α1T (green) and membrane-tethered mCherry (magenta) driven by *Ca-α1T^{Gal4}* are colocalized throughout the brain of *GFP::Ca-α1T/Ca-α1T^{Gal4};;UAS-mCD8-ChRFP/+* flies. Maximum intensity projections of **(a1)** GFP-tagged Ca-α1T, **(a2)** *Ca-α1T^{Gal4}*-driven *mCD8-ChRFP*, **(a3)** and a merged image. **(b-c)** Location corresponds to the boxed areas in (a). **(b)** Expression in the ellipsoid body (EB), fan-shaped body (FB), and noduli (NO). **(c)** Expression in the protocerebral bridge (PB), the mushroom body (MB) calyx, and the MB Kenyon cells.

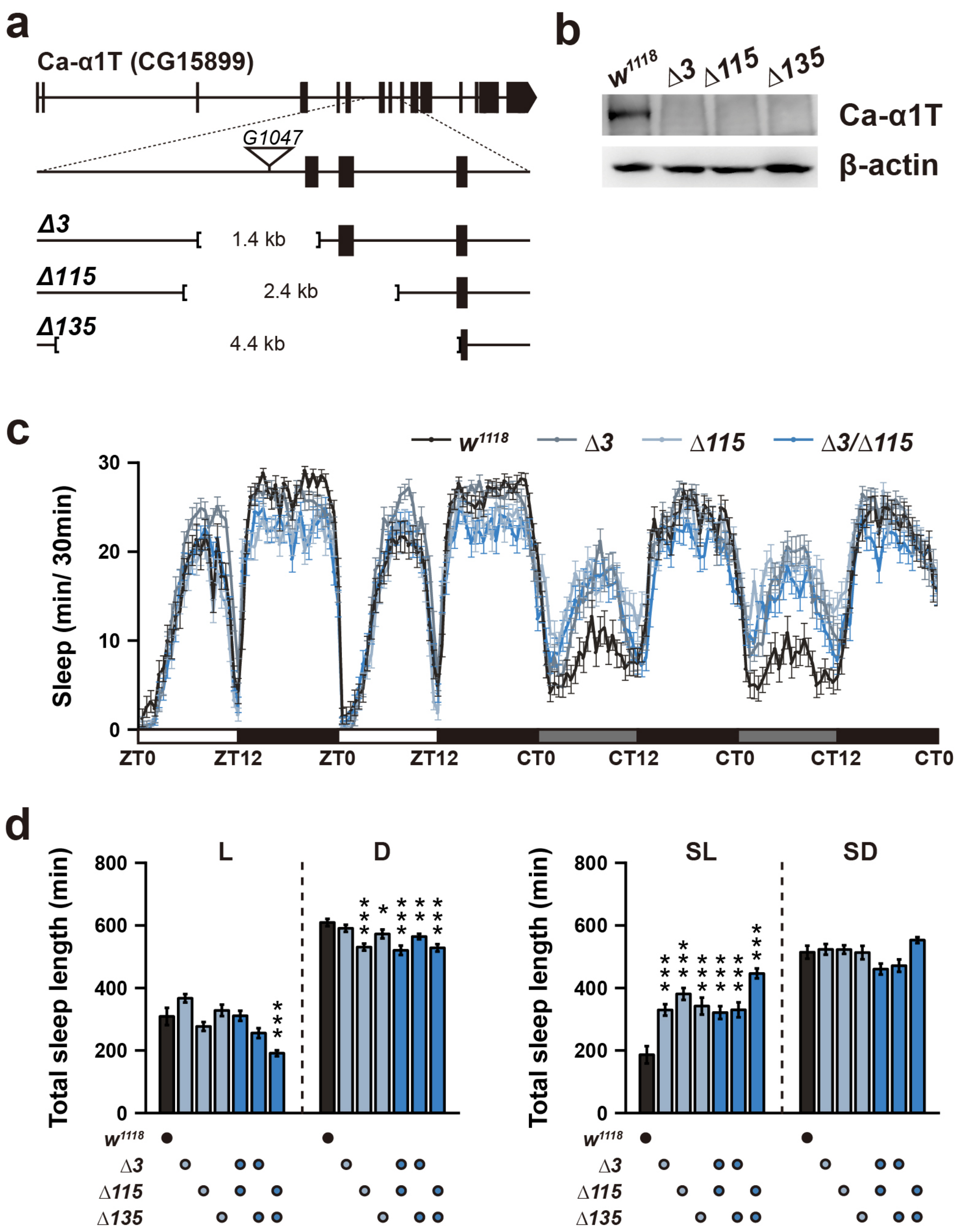

Supplementary Figure S3. Loss of Ca-α1T increases sleep in constant darkness.
(a) The Ca-α1T locus indicating the regions deleted in the Δ*3*, Δ*115* and Δ*135* mutants. The insertion point of the EP line (G1047) is indicated. Ca-α1T exons are represented in black. The bracketed areas represent the deleted regions for each mutant and the deletion sizes are indicated. **(b)** Western blot analysis of Ca-α1T protein levels in w^{1118}, Δ*3*, Δ*115* and Δ*135*. Ca-α1T protein is undetectable in lysates from all 3 deletion mutants. β-actin was used as a loading control. **(c)** Sleep profiles of w^{1118} (n=31), Δ*3* (n=31), Δ*115* (n=32) and transhetero mutant Δ*3*/Δ*115* (n=32) over two days of 12h:12h light-dark cycles (LD) and two days of continuous darkness (DD). Sleep is plotted in 30 minute intervals. White, black, and grey bars denote light phase, dark phase and subjective light phase, respectively. ZT, zeitgeber time. CT, circadian time. **(d)** Average total sleep of *Ca-α1T* mutants in the light (L) and dark phases (D) over two days of LD (left) and in the subjective light (SL) and subjective dark (SD) phases over two days of DD (right). w^{1118} (n=31), Δ*3* (n=31), Δ*115* (n=32), Δ*135* (n=29), Δ*3*/Δ*115* (n=32), Δ*3*/Δ*135* (n=32), and Δ*115*/Δ*135* (n=30). Data are presented as means ± s.e.m.. Significance was determined with Welch's t-test. *$p<0.05$, **$p<0.01$, ***$p<0.001$.

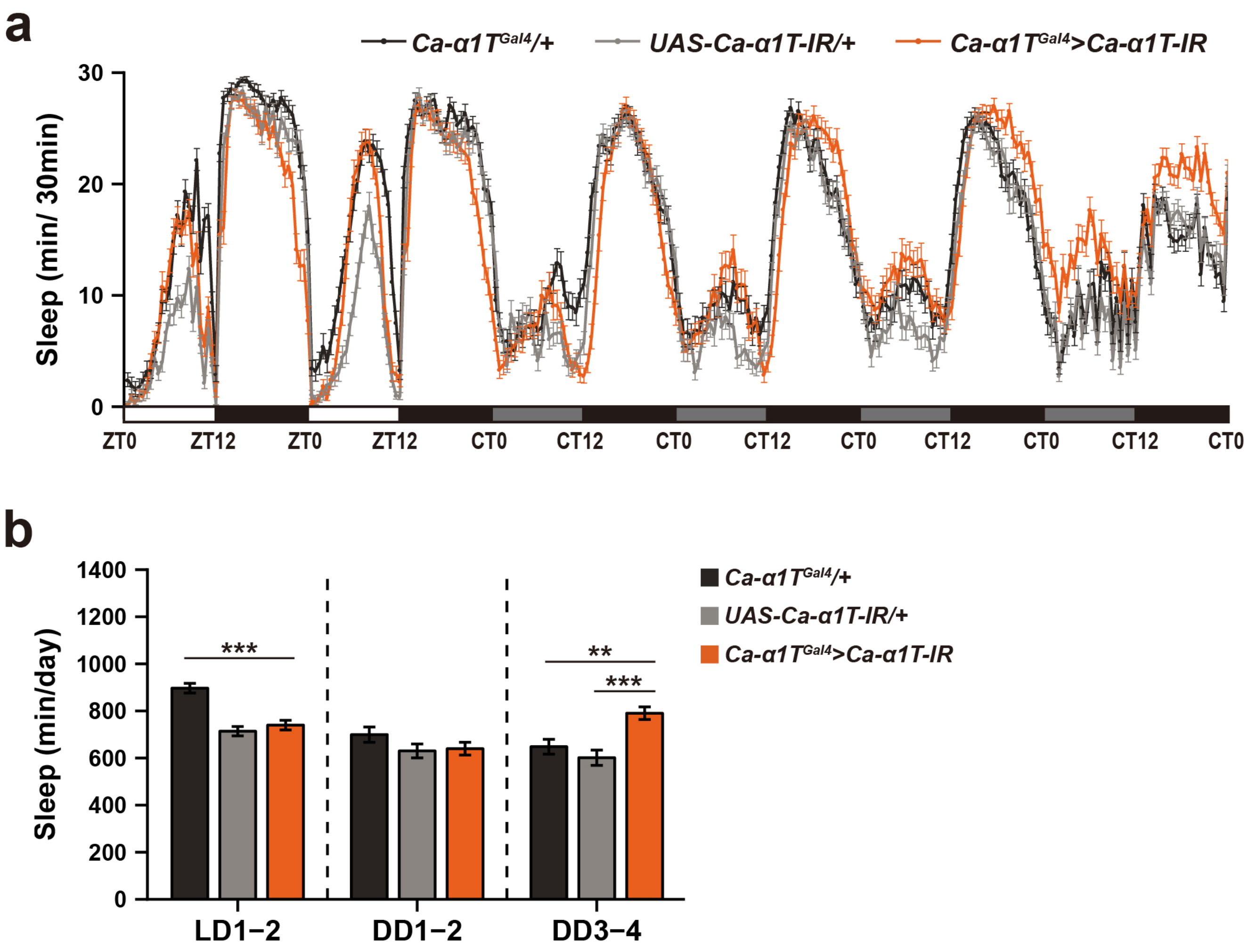

Supplementary Figure S4. ***Knock-down of Ca-α1T with the* Ca-α1T^{Gal4} driver increases sleep in constant darkness.** **(a)** Sleep profiles of *Ca-α1T^{Gal4}/+* (black, n=63), *UAS-Ca-α1T-IR/+* (grey, n=63) and *Ca-α1T^{Gal4}>Ca-α1T-IR* (orange, n=59) over two days of 12h:12h light-dark cycle (LD) and four days of continuous darkness (DD). Sleep is plotted in 30 minute intervals. White, black, and grey bars denote light phase, dark phase, and subjective light phase, respectively. ZT, zeitgeber time. CT, circadian time. **(b)** Quantification of the average total sleep from (a). Data are presented as means ± s.e.m.. Significance was determined with the one-way ANOVA followed by Tukey-HSD post hoc tests. $^{**}p<0.01$, $^{***}p<0.001$.

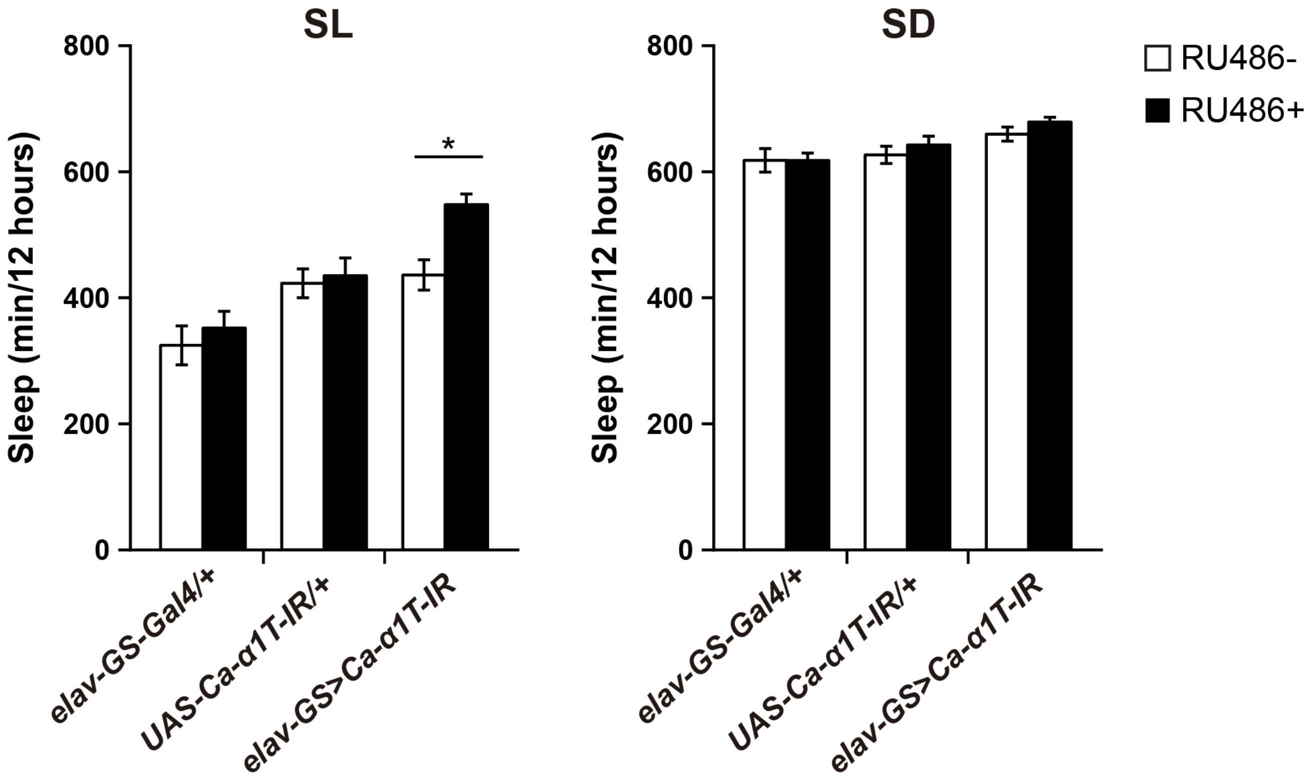

Supplementary Figure S5. Adult stage-specific knock-down of Ca-α1T increases sleep in constant darkness. Sleep during subjective daytime and subjective nighttime in continuous darkness (DD). Flies fed RU486 as adults (*elav-GS*>*Ca-α1T-IR*, black) sleep significantly longer during subjective daytime in the day 2 of DD when compared to RU486-unfed controls (*elav-GS*>*Ca-α1T-IR*, white). SL, subjective light-phase. SD, subjective dark-phase. Data are presented as means ± s.e.m.. Significance was determined using the one-way ANOVA followed by Tukey-HSD post hoc tests. *p<0.05.
